# Supplementary figures and images for: Functional classification and validation of yeast prenylation motifs using machine learning and genetic reporters
Source: PLoS One. 2022 Jun 24;17(6):e0270128. doi: 10.1371/journal.pone.0270128 (PMC9231725; doi:10.1371/journal.pone.0270128)

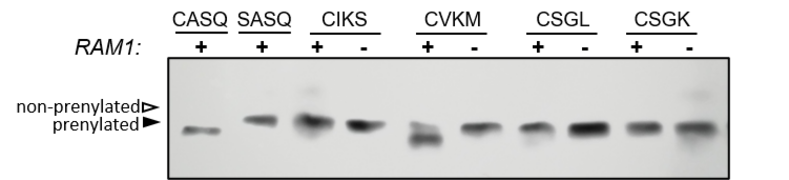

Supplement: S1 Fig — Yeast strains lacking chromosomally encoded YDJ1 +/- RAM1 (yWS304, ydj1Δ or yWS2542, ydj1Δram1Δ) expressing Ydj1p-Cxxx plasmids of sequences were evaluated in the presence/absence of FTase (RAM1 gene) as described in Fig 3. Sequences were selected from Fig 3A for further evaluation due to unclear gel shift or prenylation status. (TIF) [file pone.0270128.s001.tif]
