# Supplementary material for: Functional classification and validation of yeast prenylation motifs using machine learning and genetic reporters
Source: PLoS One. 2022 Jun 24;17(6):e0270128. doi: 10.1371/journal.pone.0270128 (PMC9231725; doi:10.1371/journal.pone.0270128)
Supplement: S1 Table — (DOCX) [file pone.0270128.s002.docx]

**S1 Table. Probability estimates and prediction calls for prenylation and cleavage of naturally occurring yeast Cxxx sequences as reported by the SVM-ESM-1b model.**

|  |  | prenylation | | cleavage | |
| --- | --- | --- | --- | --- | --- |
| yeast protein | **motif** | **score^a^** | **prediction** | **score** | **prediction** |
| Ras2 | CIIS | 0.9969 | + | 6.1594 | + |
| Hmg1 | CIKS | 0.2587 | - | NA | NA |
| Rho2 | CIIL | 0.9880 | + | 5.0875 | + |
| Ssp2 | CIDL | 0.0534 | - | NA | NA |
| Skt5, MiY1 | CVIM | 1.0000 | + | 8.9377 | + |
| Tbs1 | CVKM | 0.1811 | -**^b^** | 1.8026 | - |
| YDL022C-A | CSII | 0.9762 | + | 5.9957 | + |
| YBR096W | CSEI | 0.1155 | - | NA | NA |
| YMR265C | CSNA | 0.1339 | -**^b^** | -0.6976 | - |
| Pet18 | CYNA | 0.0738 | -**^b^** | -0.2218 | - |
| Lih1 | CSGL | 0.1847 | - | NA | NA |
| Cup1 | CSGK | 0.0034 | - | NA | NA |
| Nap1 | CKQS | 0.5680 | + | 1.8671 | - |
| Cst26 | CFIF | 0.9800 | + | 5.1732 | +**^b^** |
| YIL134C-A | CAPY | 0.6833 | + | -1.2698 | - |
| Atr1 | CTVA | 0.9860 | + | 4.8219 | + |
| Las21 | CALD | 0.6017 | + | 2.4882 | - |
| YDL009C | CAVS | 0.9831 | + | 4.3306 | + |
| Sua5 | CIQF | 0.9018 | + | 4.0671 | - |

**^a^**Probability estimates were determined for the SVM-ESM-1b model using Platt-scaling. Signs represent predictions of prenylation and that were reported as positive (+) or negative (-) by the SVM-ESM-1b model. NA – not applicable.

**^b^**Prediction differs from empirical observation.
