# Supplementary material for: Functional classification and validation of yeast prenylation motifs using machine learning and genetic reporters
Source: PLoS One. 2022 Jun 24;17(6):e0270128. doi: 10.1371/journal.pone.0270128 (PMC9231725; doi:10.1371/journal.pone.0270128)
Supplement: S2 Table — (DOCX) [file pone.0270128.s003.docx]

**S2 Table. Prediction calls for cleavage of naturally occurring yeast Cxxx sequences by indicated model**.

| yeast protein | motif | PSSM^a,b^ | Naïve Bayes | kNN |
| --- | --- | --- | --- | --- |
| Ras2 | CIIS | + | + | + |
| Hmg1 | CIKS | NA | NA | NA |
| Rho2 | CIIL | + | + | + |
| Ssp2 | CIDL | NA | NA | NA |
| Skt5, MiY1 | CVIM | + | + | + |
| Tbs1 | CVKM | - | - | - |
| YDL022C-A | CSII | + | + | + |
| YBR096W | CSEI | NA | NA | NA |
| YMR265C | CSNA | - | - | - |
| Pet18 | CYNA | - | - | - |
| Lih1 | CSGL | NA | NA | NA |
| Cup1 | CSGK | NA | NA | NA |
| Nap1 | CKQS | - | - | - |
| Cst26 | CFIF | +**^c^** | +**^c^** | +**^c^** |
| YIL134C-A | CAPY | - | - | - |
| Atr1 | CTVA | + | + | + |
| Las21 | CALD | - | - | - |
| YDL009C | CAVS | -**^c^** | -**^c^** | -**^c^** |
| Sua5 | CIQF | - | - | - |
| number observed/predicted | | 12/14 | 12/14 | 12/14 |
| % observed/predicted | | 85.7 | 85.7 | 85.7 |
|  | |  |  |  |
| number false positive | | 1/7 | 1/7 | 1/7 |
| % false positive | | 14.3 | 14.3 | 14.3 |
|  | |  |  |  |
| number false negative | | 1/7 | 1/7 | 1/7 |
| % false negative | | 14.3 | 14.3 | 14.3 |

**^a^**PSSM – PSSM sequence; Naïve Bayes – Naïve Bayes AAindex; kNN – kNN sequence.

**^b^**Signs represent predictions of cleavage that were reported as positive (+) or negative (-) by the indicated model. NA – not applicable.

**^c^**Prediction differs from empirical observation.
