# Supplementary material for: Functional classification and validation of yeast prenylation motifs using machine learning and genetic reporters
Source: PLoS One. 2022 Jun 24;17(6):e0270128. doi: 10.1371/journal.pone.0270128 (PMC9231725; doi:10.1371/journal.pone.0270128)
Supplement: S3 Table — (DOCX) [file pone.0270128.s004.docx]

**S3 Table. Yeast strains used in this study.**

| **Strain** | **Genotype** | **Reference** |
| --- | --- | --- |
| BY4741 | *MAT***a** *his3∆1 leu2∆0 met15∆0 ura3∆0* | [1] |
| IH1793; ATCC#204279 | *MAT*α *lys1* | [2] |
| SM2331 | *MAT***a** *trp1 leu2 ura3 his4 can1 mfa1-∆1 mfa2-∆1* | [3] |
| yWS164 | *MAT***a** *trp1 leu2 ura3 his4 can1 mfa1-∆1 mfa2-∆1 rce1::TRP1 ste24::KAN^R^* | [4] |
| yWS304 | *MAT***a** *his3∆1 leu2∆0 met15∆0 ura3∆0 ydj1∆::KAN^R^* | [5] |
| yWS2393 | *MAT***a** *trp1 leu2 ura3 his4 can1 mfa1-∆1 mfa2-∆1 ste24::KAN^R^* | This study |
| yWS2462 | *MAT***a** *trp1 leu2 ura3 his4 can1 mfa1-∆1 mfa2-∆1*  *rce1::KAN^R^* | This study |
| yWS2542 | *MATa his3 leu2 met15 ura3 ydj1∆::NAT^R^ ram1∆::KAN^R^* | [6] |
| yWS2544 | *MAT***a** *his3∆1 leu2∆0 met15∆0 ura3∆0 ydj1∆::NAT^R^* | [6] |

**References**

1. Shoemaker DD, Lashkari DA, Morris D, Mittmann M, Davis RW. Quantitative phenotypic analysis of yeast deletion mutants using a highly parallel molecular bar-coding strategy. Nature genetics. 1996;14(4):450-6.

2. Michaelis S, Herskowitz I. The a-factor pheromone of Saccharomyces cerevisiae is essential for mating. Molecular and cellular biology. 1988;8(3):1309-18.

3. Chen P, Sapperstein SK, Choi JD, Michaelis S. Biogenesis of the Saccharomyces cerevisiae mating pheromone a-factor. The Journal of cell biology. 1997;136(2):251-69.

4. Cadiñanos J, Schmidt WK, Fueyo A, Varela I, López-Otín C, Freije JMP. Identification, functional expression and enzymic analysis of two distinct CaaX proteases from Caenorhabditis elegans. The Biochemical journal. 3702003. p. 1047-54.

5. Giaever G, Chu AM, Ni L, Connelly C, Riles L, Véronneau S, et al. Functional profiling of the Saccharomyces cerevisiae genome. Nature. 2002;418(6896):387-91.

6. Berger BM, Kim JH, Hildebrandt ER, Davis IC, Morgan MC, Hougland JL, et al. Protein Isoprenylation in Yeast Targets COOH-Terminal Sequences Not Adhering to the CaaX Consensus. Genetics. 2018;210(4):1301-16.
