# Supplementary material for: Functional classification and validation of yeast prenylation motifs using machine learning and genetic reporters
Source: PLoS One. 2022 Jun 24;17(6):e0270128. doi: 10.1371/journal.pone.0270128 (PMC9231725; doi:10.1371/journal.pone.0270128)
Supplement: S4 Table — (DOCX) [file pone.0270128.s005.docx]

**S4 Table. Yeast expression plasmids used in this study.**

| Gene | Identifier | Genotype | Reference |
| --- | --- | --- | --- |
| vectors | pRS315 | *CEN LEU2* | [1] |
|  | pRS316 | *CEN URA3* | [1] |
|  | pRS415 | *CEN LEU2* | [1] |
| *HsRce1* | pWS130 | *2µ URA3 P_PGK_ HsRce1∆22* | This study |
| *HsSte24* | pWS1609 | *CEN URA3 P_PGK_ HsSTE24* | This study |
| *YDJ1* | pWS942 | *CEN URA3 YDJ1* | [2] |
|  | pWS1132 | *CEN URA3 YDJ1-SASQ* | [2] |
|  | pWS1343 | *CEN URA3 YDJ1-CASA* | [3] |
|  | pWS1372 | *CEN URA3 YDJ1-CAAQ* | [3] |
|  | pWS1410 | *CEN URA3 YDJ1-CVAA* | This study |
|  | pWS1411 | *CEN URA3 YDJ1-CKQS* | This study |
|  | pWS1437 | *CEN URA3 YDJ1-CAHQ* | This study |
|  | pWS1456 | *CEN URA3 YDJ1-CAKS* | This study |
|  | pWS1460 | *CEN URA3 YDJ1-CQTS* | This study |
|  | pWS1461 | *CEN URA3 YDJ1-CSFL* | This study |
|  | pWS1463 | *CEN URA3 YDJ1-CVIM* | This study |
|  | pWS1729 | *CEN URA3 YDJ1-CTDS* | This study |
|  | pWS1745 | *CEN URA3 YDJ1-CALD* | This study |
|  | pWS1746 | *CEN URA3 YDJ1-CAPY* | This study |
|  | pWS1747 | *CEN URA3 YDJ1-CAVS* | This study |
|  | pWS1748 | *CEN URA3 YDJ1-CFIF* | This study |
|  | pWS1749 | *CEN URA3 YDJ1-CIDL* | This study |
|  | pWS1751 | *CEN URA3 YDJ1-CIIL* | This study |
|  | pWS1752 | *CEN URA3 YDJ1-CIKS* | This study |
|  | pWS1753 | *CEN URA3 YDJ1-CIQF* | This study |
|  | pWS1757 | *CEN URA3 YDJ1-CSEI* | This study |
|  | pWS1758 | *CEN URA3 YDJ1-CSGK* | This study |
|  | pWS1759 | *CEN URA3 YDJ1-CSGL* | This study |
|  | pWS1760 | *CEN URA3 YDJ1-CSII* | This study |
|  | pWS1761 | *CEN URA3 YDJ1-CSNA* | This study |
|  | pWS1762 | *CEN URA3 YDJ1-CTVA* | This study |
|  | pWS1763 | *CEN URA3 YDJ1-CVKM* | This study |
|  | pWS1764 | *CEN URA3 YDJ1-CYNA* | This study |
|  | pWS1830 | *CEN URA3 YDJ1-CNLI* | This study |
|  | pWS1834 | *CEN URA3 YDJ1-CVFM* | This study |
|  | pWS2021 | *CEN URA3 YDJ1-CKQG* | This study |
|  | pWS2022 | *CEN URA3 YDJ1-CKQH* | This study |
|  | pWS2025 | *CEN URA3 YDJ1-CKQL* | This study |
| *MFA1* | pWS610 | *CEN LEU2 MFA1* | [4] |
|  | pWS613 | *CEN LEU2 MFA1-CTLM* | [2] |
|  | pWS846 | *CEN LEU2 MFA1-CKQS* | [4] |
|  | pWS1561 | *CEN LEU2 MFA1-CSIM* | This study |
|  | pWS1562 | *CEN LEU2 MFA1-CIIS* | This study |
|  | pWS1671 | *CEN LEU2 MFA1-CTVA* | This study |
|  | pWS1730 | *CEN LEU2 MFA1-CALD* | This study |
|  | pWS1733 | *CEN LEU2 MFA1-CIQF* | This study |
|  | pWS1734 | *CEN LEU2 MFA1-CYNA* | This study |
|  | pWS1738 | *CEN LEU2 MFA1-CVIM* | This study |
|  | pWS1739 | *CEN LEU2 MFA1-CSGL* | This study |
|  | pWS1777 | *CEN LEU2 MFA1-CAVS* | This study |
|  | pWS1778 | *CEN LEU2 MFA1-CFIF* | This study |
|  | pWS1779 | *CEN LEU2 MFA1-CIIL* | This study |
|  | pWS1780 | *CEN LEU2 MFA1-CIKS* | This study |
|  | pWS1781 | *CEN LEU2 MFA1-CIDL* | This study |
|  | pWS1782 | *CEN LEU2 MFA1-CSII* | This study |
|  | pWS1783 | *CEN LEU2 MFA1-CSEI* | This study |
|  | pWS1784 | *CEN LEU2 MFA1-CSNA* | This study |
|  | pWS1785 | *CEN LEU2 MFA1-CAPY* | This study |
|  | pWS1788 | *CEN LEU2 MFA1-CSGK* | This study |
|  | pWS1809 | *CEN LEU2 MFA1-CVKM* | This study |

**References**

1. Sikorski RS, Hieter P. A system of shuttle vectors and yeast host strains designed for efficient manipulation of DNA in Saccharomyces cerevisiae. Genetics. 1989;122(1):19-27.

2. Hildebrandt ER, Cheng M, Zhao P, Kim JH, Wells L, Schmidt WK. A shunt pathway limits the CaaX processing of Hsp40 Ydj1p and regulates Ydj1p-dependent phenotypes. eLife. 2016;5.

3. Berger BM, Kim JH, Hildebrandt ER, Davis IC, Morgan MC, Hougland JL, et al. Protein Isoprenylation in Yeast Targets COOH-Terminal Sequences Not Adhering to the CaaX Consensus. Genetics. 2018;210(4):1301-16.

4. Krishnankutty RK, Kukday SS, Castleberry AJ, Breevoort SR, Schmidt WK. Proteolytic processing of certain CaaX motifs can occur in the absence of the Rce1p and Ste24p CaaX proteases. Yeast (Chichester, England). 2009;26(8):451-63.
