# Supplementary material for: Functional classification and validation of yeast prenylation motifs using machine learning and genetic reporters
Source: PLoS One. 2022 Jun 24;17(6):e0270128. doi: 10.1371/journal.pone.0270128 (PMC9231725; doi:10.1371/journal.pone.0270128)
Supplement: S5 Table — (DOCX) [file pone.0270128.s006.docx]

**S5 Table. PCR Oligonucleotides used in this study.**

| **Gene** | **Oligo** | **Mutation** | **Sequence (5' to 3')** |
| --- | --- | --- | --- |
| Plasmid UTR | oWS219^a^ | NA^b^ | tgaCCATGATTACGCCAAGC |
| *YDJ1* | oWS999 | CVAA | TCCGATGAAGAAGAACAAGGTGGCGAAGGTGTTCAATGTGTTGCCGCATGAttttcttgataaaaaaagatca |
|  | oWS1000 | CKQS | TCCGATGAAGAAGAACAAGGTGGCGAAGGTGTTCAATGTAAGCAGAGCTGAttttcttgataaaaaaagatca |
|  | oWS1008 | CAHQ | GATTCCGATGAAGAAGAACAAGGTGGCGAAGGTGTTCAATGTGCTCAtCAATGAttttcttgataaaaaaagatcaac |
|  | oWS1028 | CIIS | GATTCCGATGAAGAAGAACAAGGTGGCGAAGGTGTTCAATGTatcatttctTGAttttcttgataaaaaaagatca |
|  | oWS1032 | CQTS | GATTCCGATGAAGAAGAACAAGGTGGCGAAGGTGTTCAATGTcaaacatctTGAttttcttgataaaaaaagatca |
|  | oWS1033 | CSFL | GATTCCGATGAAGAAGAACAAGGTGGCGAAGGTGTTCAATGTtcttttttgTGAttttcttgataaaaaaagatca |
|  | oWS1035 | CVIM | GATTCCGATGAAGAAGAACAAGGTGGCGAAGGTGTTCAATGTgttatcatgTGAttttcttgataaaaaaagatca |
|  | oWS1051 | NA^b^ | GGTATGAAGTGGAGGGAGGAT |
|  | oWS1334 | CALD | TCCGATGAAGAAGAACAAGGTGGCGAAGGTGTTCAATGTGCTTTGGATTGAttttcttgataaaaaaagatc |
|  | oWS1335 | CAPY | TCCGATGAAGAAGAACAAGGTGGCGAAGGTGTTCAATGTGCTCCATATTGAttttcttgataaaaaaagatc |
|  | oWS1336 | CAVS | TCCGATGAAGAAGAACAAGGTGGCGAAGGTGTTCAATGTGCTGTTTCTTGAttttcttgataaaaaaagatc |
|  | oWS1337 | CFIF | TCCGATGAAGAAGAACAAGGTGGCGAAGGTGTTCAATGTTTTATTTTTTGAttttcttgataaaaaaagatc |
|  | oWS1338 | CIDL | TCCGATGAAGAAGAACAAGGTGGCGAAGGTGTTCAATGTATTGATTTGTGAttttcttgataaaaaaagatc |
|  | oWS1340 | CIIL | TCCGATGAAGAAGAACAAGGTGGCGAAGGTGTTCAATGTATTATTTTGTGAttttcttgataaaaaaagatc |
|  | oWS1341 | CIKS | TCCGATGAAGAAGAACAAGGTGGCGAAGGTGTTCAATGTATTAAATCTTGAttttcttgataaaaaaagatc |
|  | oWS1342 | CIQF | TCCGATGAAGAAGAACAAGGTGGCGAAGGTGTTCAATGTATTCAATTTTGAttttcttgataaaaaaagatc |
|  | oWS1346 | CSEI | TCCGATGAAGAAGAACAAGGTGGCGAAGGTGTTCAATGTTCTGAAATTTGAttttcttgataaaaaaagatc |
|  | oWS1347 | CSGK | TCCGATGAAGAAGAACAAGGTGGCGAAGGTGTTCAATGTTCTGGTAAATGAttttcttgataaaaaaagatc |
|  | oWS1348 | CSGL | TCCGATGAAGAAGAACAAGGTGGCGAAGGTGTTCAATGTTCTGGTTTGTGAttttcttgataaaaaaagatc |
|  | oWS1349 | CSII | TCCGATGAAGAAGAACAAGGTGGCGAAGGTGTTCAATGTTCTATTATTTGAttttcttgataaaaaaagatc |
|  | oWS1350 | CSNA | TCCGATGAAGAAGAACAAGGTGGCGAAGGTGTTCAATGTTCTAATGCTTGAttttcttgataaaaaaagatc |
|  | oWS1351 | CTVA | TCCGATGAAGAAGAACAAGGTGGCGAAGGTGTTCAATGTACTGTTGCTTGAttttcttgataaaaaaagatc |
|  | oWS1352 | CVKM | TCCGATGAAGAAGAACAAGGTGGCGAAGGTGTTCAATGTGTTAAAATGTGAttttcttgataaaaaaagatc |
|  | oWS1353 | CYNA | TCCGATGAAGAAGAACAAGGTGGCGAAGGTGTTCAATGTTATAATGCTTGAttttcttgataaaaaaagatc |
|  | oWS1423 | CNLI | TCCGATGAAGAAGAACAAGGTGGCGAAGGTGTTCAATGTAATTTGATTTGAttttcttgataaaaaaagatc |
|  | oWS1444 | CVFM | TCCGATGAAGAAGAACAAGGTGGCGAAGGTGTTCAATGTGTTTTTATGTGAttttcttgataaaaaaagatc |
|  | oWS1583 | CKQG | tccgatgaagaagaacaaggtggcgaaggtgttcaatgtAAACAAGGTtgattttcttgataaaaaaagatc |
|  | oWS1584 | CKQH | tccgatgaagaagaacaaggtggcgaaggtgttcaatgtAAACAACATtgattttcttgataaaaaaagatc |
|  | oWS1587 | CKQL | tccgatgaagaagaacaaggtggcgaaggtgttcaatgtAAACAATTGtgattttcttgataaaaaaagatc |
| *MFA1* | oWS356 | CVKM | AACTATATTATCAAAGGTGTCTTCTGGGACCCAGCATGcgtaaaaatgTAGTTTCTGCGTACAAAAACGCGT |
|  | oWS1178 | CSIM | AACTATATTATCAAAGGTGTCTTCTGGGACCCAGCATGctctatcatgTAGTTTCTGCGTACAAAAACGCGT |
|  | oWS1179 | CIIS | AACTATATTATCAAAGGTGTCTTCTGGGACCCAGCATGCatcatttctTAGTTTCTGCGTACAAAAACGCGT |
|  | oWS1224 | CALD | AACTATATTATCAAAGGTGTCTTCTGGGACCCAGCATGTgctttggatTAGtttctgcgtacaaaaacgCGT |
|  | oWS1232 | CIIL | AACTATATTATCAAAGGTGTCTTCTGGGACCCAGCATGTattattttgTAGtttctgcgtacaaaaacgCGT |
|  | oWS1234 | CIQF | AACTATATTATCAAAGGTGTCTTCTGGGACCCAGCATGTattcaatttTAGtttctgcgtacaaaaacgCGT |
|  | oWS1236 | CSGL | AACTATATTATCAAAGGTGTCTTCTGGGACCCAGCATGTtctggtttgTAGtttctgcgtacaaaaacgCGT |
|  | oWS1239 | CTVA | AACTATATTATCAAAGGTGTCTTCTGGGACCCAGCATGTactgttgctTAGtttctgcgtacaaaaacgCGT |
|  | oWS1241 | CVIM | AACTATATTATCAAAGGTGTCTTCTGGGACCCAGCATGTgttattatgTAGtttctgcgtacaaaaacgCGT |
|  | oWS1244 | CYNA | AACTATATTATCAAAGGTGTCTTCTGGGACCCAGCATGTtataatgctTAGtttctgcgtacaaaaacgCGT |
|  | oWS1357 | CAVS | AACTATATTATCAAAGGTGTCTTCTGGGACCCAGCATGTgctgtttctTAGtttctgcgtacaaaaacgCGT |
|  | oWS1358 | CFIF | AACTATATTATCAAAGGTGTCTTCTGGGACCCAGCATGTtttatttttTAGtttctgcgtacaaaaacgCGT |
|  | oWS1362 | CIKS | AACTATATTATCAAAGGTGTCTTCTGGGACCCAGCATGcattaaatctTAGtttctgcgtacaaaaacgCGT |
|  | oWS1363 | CIDL | AACTATATTATCAAAGGTGTCTTCTGGGACCCAGCATGcattgatttgTAGtttctgcgtacaaaaacgCGT |
|  | oWS1364 | CSII | AACTATATTATCAAAGGTGTCTTCTGGGACCCAGCATGctctattattTAGtttctgcgtacaaaaacgCGT |
|  | oWS1365 | CSEI | AACTATATTATCAAAGGTGTCTTCTGGGACCCAGCATGctctgaaattTAGtttctgcgtacaaaaacgCGT |
|  | oWS1366 | CSNA | AACTATATTATCAAAGGTGTCTTCTGGGACCCAGCATGctctaatgctTAGtttctgcgtacaaaaacgCGT |
|  | oWS1367 | CSGK | AACTATATTATCAAAGGTGTCTTCTGGGACCCAGCATGctctggtaaaTAGtttctgcgtacaaaaacgCGT |
|  | oWS1369 | CAPY | AACTATATTATCAAAGGTGTCTTCTGGGACCCAGCATGcgctccttatTAGtttctgcgtacaaaaacgCGT |

**^a^**Reverse oligonucleotide paired with listed *MFA1* or *YDJ1* mutagenic oligonucleotides.

**^b^**Not applicable.

**^c^**Lowercase letters indicate differences from wildtype sequences.
