# Supplementary material for: Functional classification and validation of yeast prenylation motifs using machine learning and genetic reporters
Source: PLoS One. 2022 Jun 24;17(6):e0270128. doi: 10.1371/journal.pone.0270128 (PMC9231725; doi:10.1371/journal.pone.0270128)
Supplement: S1 Raw images — (PDF) [file pone.0270128.s010.pdf]

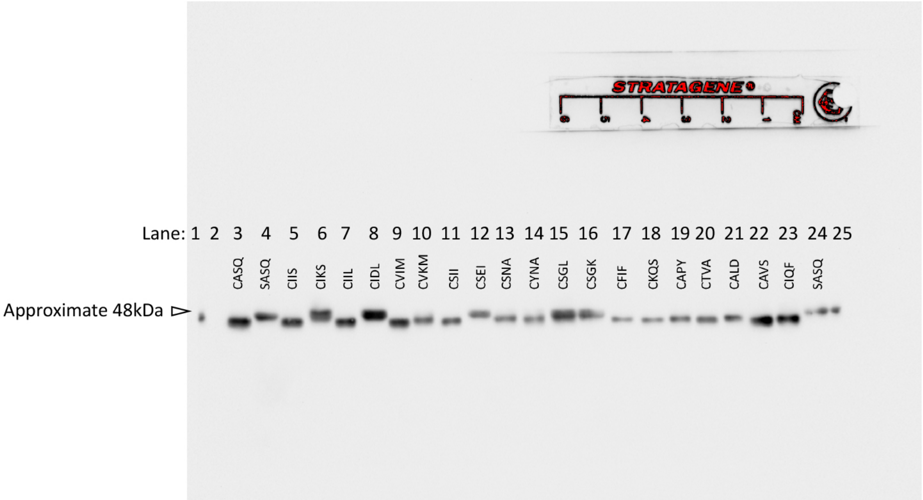

Fig 4A: Prenylation Gel Shift of Ydj1p-Cxxx variants.
Blot was imaged by Bio-Rad ChemiDoc Imaging system. 48 kDa marker from FroggaBio BLUelf Prestained Protein ladder is indicated.

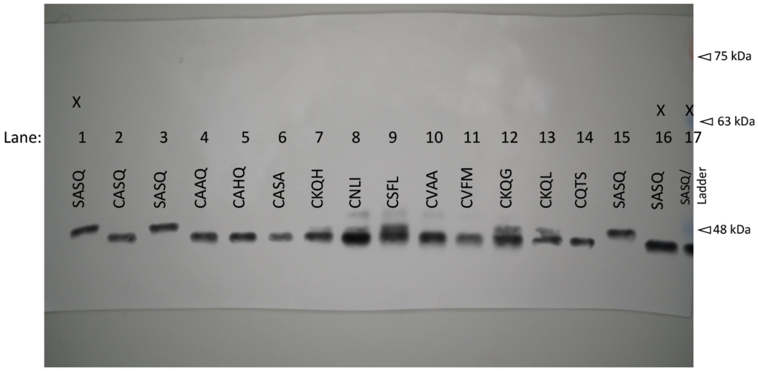

Fig 4B: Prenylation Gel Shift of Ydj1p-Cxxx variants. Blot was imaged using Kwikquant digital imager (Kindle Biosciences, Greenwich, Connecticut). Membrane was trimmed through Lane 17 containing extra sample/ladder before imaging in order to fit within imaging apparatus.

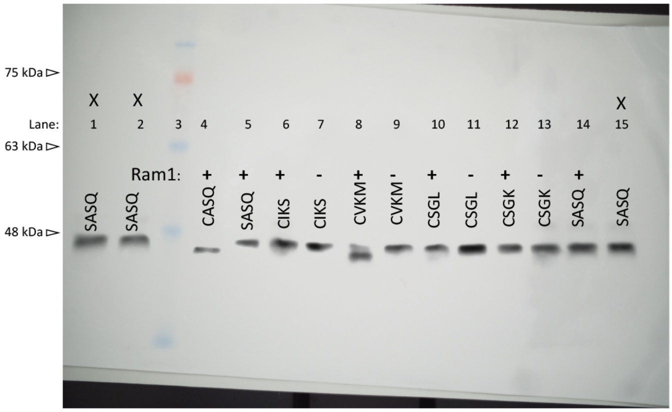

Fig S1: Prenylation Gel Shift of Ydj1p-Cxxx variants. Blot was imaged using Kwikquant digital imager (Kindle Biosciences, Greenwich, Connecticut)
